# Hypertensive disorders of pregnancy may increase the risk of neurodevelopmental delay in the offspring: a systematic review with meta-analysis

**DOI:** 10.1038/s41440-026-02750-1

**Published:** 2026-08-03

**Authors:** Leslie Marilu Zavala-Martínez, Erika Marlen Hurtado-Salgado, Mary Carmen Baltazar-Reyes, Elizabeth Guzmán-Ortiz, Lea Aurora Cupul-Uicab

**Affiliations:** 1 Division of Health Sciences and Engineering, University of Guanajuato, Campus Celaya-Salvatierra. Av. Ing. Javier Barrios Sierra 201 Esq. Av. Baja California, Ejido de Santa María del Refugio, Celaya, Guanajuato México; 2 Center for Population Health Research, National Institute of Public Health. Avenida Universidad 655, Colonia Santa María Ahuacatlán, Cuernavaca, Morelos México

**Keywords:** implemental hypertension, hypertensive disorders of pregnancy, meta-analysis, neurodevelopment, offspring

## Abstract

Hypertensive disorders of pregnancy (HDP) are the most frequent complication of pregnancy, posing a burden on maternal and offspring health. Evidence about the potential effect of HDP on the infants’ neurodevelopment is still inconsistent. We summarize the evidence about the effect of HDP on neurodevelopmental delay in the offspring. We conducted a systematic review with meta-analysis following the PRISMA (Preferred Reporting Items for Systematic reviews with Meta-Analysis) guidelines. The search was conducted in PubMed, Web of Science, and EBSCO databases. 21 publications were selected (20 cohorts and one case-control); 13/21 had data to meta-analyze. Children exposed to HDP doubled their risk of global neurodevelopment delay (OR = 2.07; 95%CI: 1.08, 3.95; *n* = 7214) compared to unexposed. Exposure to HDP was also related to a higher risk of delayed motor (OR = 1.25; 95%CI: 1.01, 1.56; *n* = 53,138) and language (OR = 1.87; 95% CI: 1.07, 3.29; *n* = 7581) development in the offspring. Considerable heterogeneity between studies was observed in meta-analyses of delayed global neurodevelopment (I^2^ = 86.9%) and language development (I^2^ = 68.9%), whereas heterogeneity was not relevant for delayed motor development (I^2^ = 13.0%). HDP could also increase the risk of cognitive development delay (OR = 1.27; 95%CI: 0.88, 1.82; I^2^ = 45.8%; *n* = 6631) in the offspring. Our results support the hypothesis that exposure to HDP might increase the risk of neurodevelopmental delay in the offspring, specifically affecting motor and language domains. Prospective studies with longer follow-up times and careful control of confounders are needed to confirm whether neurodevelopmental alterations remain at older ages, to inform decision makers about specific windows for interventions.

**Figure:**
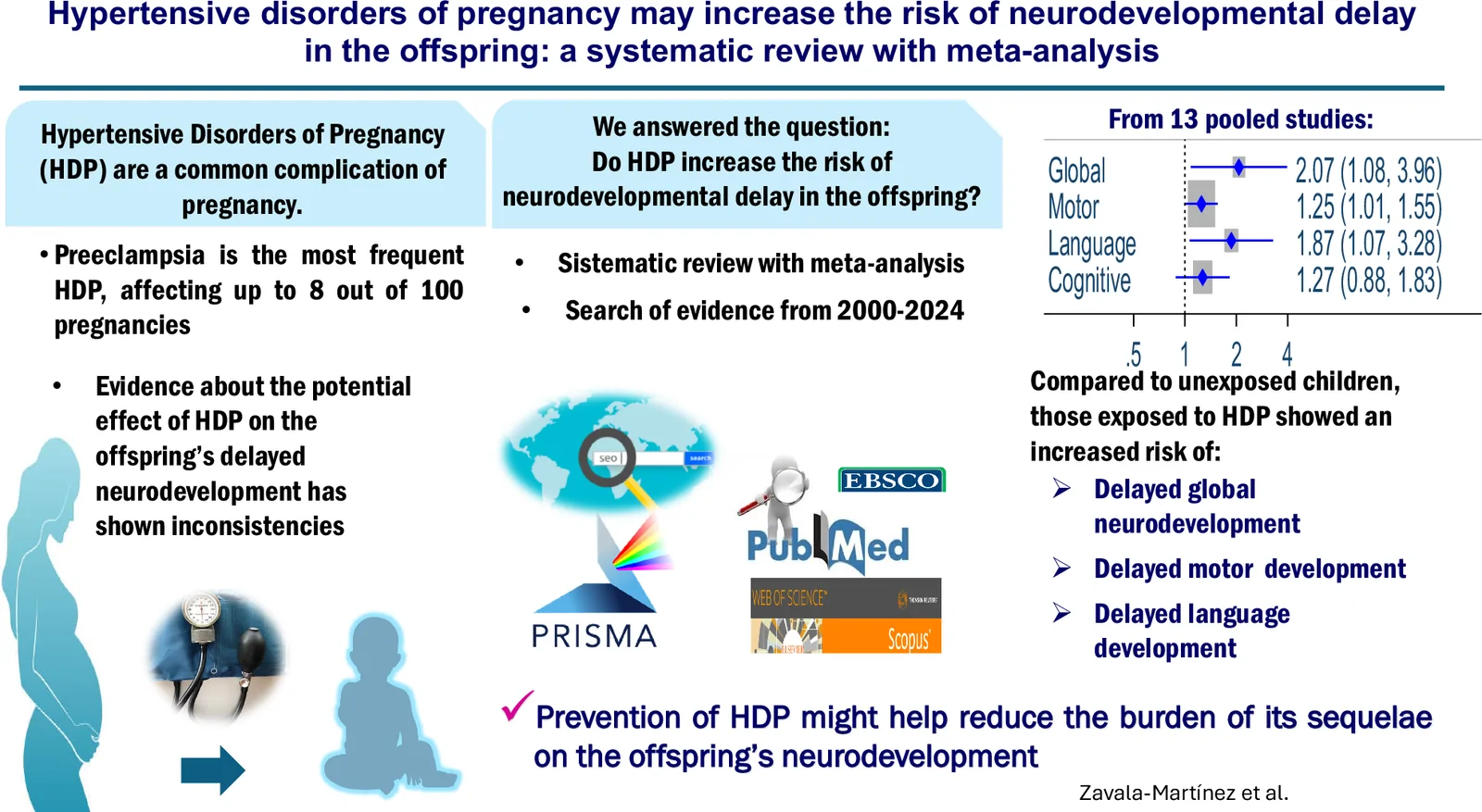

## Introduction

Hypertensive disorders of pregnancy (HDP) is a group of pathologies that include gestational hypertension (GHTN), preeclampsia (PE), eclampsia (E), and HELLP syndrome (hemolysis, elevated liver enzymes, and low platelet count); these pathologies affect approximately 10% of pregnancies worldwide [1], therefore is considered a relevant public health problem. HDP is one of the leading causes of maternal death (14–16%) [2, 3], whereas preeclampsia is the most frequent pregnancy complication with a worldwide prevalence ranging from 2% to 8% [4].

HDP has short- and long-term adverse consequences on maternal and offspring’s health. Women who develop HDP are at higher risk of having placental abruption, cerebrovascular bleeding, pregnancy loss, and death in the short term [5]; while in the long term, they are at a higher risk of developing cardiovascular and renal diseases, type 2 diabetes, and premature death from cardiovascular diseases [6–10]. In the offspring, short-term outcomes are intrauterine growth retardation, being born prematurely, low birthweight, and death [1, 11–14]. Whereas long-term effects include neurodevelopmental alterations such as cerebral palsy (CP), autism spectrum disorders (ASD), attention deficit and hyperactivity disorder (ADHD), and cognitive disorders [3, 15]. Nonetheless, the available epidemiological evidence regarding these long-term outcomes in the offspring is still inconsistent [16–20].

The biological mechanism that explains the association of HDP with neurodevelopmental alterations in the offspring is not entirely clear. However, prior evidence suggests that oxidative stress, endothelial dysfunction, systemic inflammation, and placental insufficiency caused by PE could interrupt the physiological development of the fetal brain at critical stages of gestation [21]; such alterations might be implicated in the mechanism that leads to neurodevelopmental alterations.

The global prevalence (2010–2022) of any developmental delay (i.e., motor, adaptive, cognitive, and social-emotional) in children (<12 years) from low- and middle-income countries was 18.83%, such prevalence was higher among children under five (21.2%) and in the African region (26.7%) [22]. Among US children aged 3 to 17 years, the prevalence of any developmental delay (*e.g*., ASD, ADHD, CP, blindness, hearing loss, stuttering, seizures, learning disabilities, intellectual disability, and others) was 16.8%, and among non-Hispanic African Americans was 19%; the prevalence of other developmental delays was 4.4% [23].

Children’s neurodevelopmental delay represents a significant burden for families, healthcare systems, and society, especially in the early years of life, due to the associated costs of medical care. Moreover, these conditions pose long-term implications on the emotional and social well-being of the affected individuals. Therefore, we aimed to systematically summarize and integrate evidence related to maternal hypertensive disorders of pregnancy as a potential risk factor of neurodevelopmental impairment in offspring. We focus on global neurodevelopmental delay and its specific domains, such as motor, language, and cognition, under the assumption that these outcomes are potentially modifiable as well as HDP; therefore, the potential for prevention would be higher than for any other developmental disorders.

## Methods

### Data source and search algorithms

The present review followed the PRISMA statement guidelines for the development of systematic reviews and meta-analyses (*Preferred Reporting Items for Systematic Reviews and Meta-Analyses*) [24]. We identified evidence about the association between hypertensive disorders of pregnancy and neurodevelopmental alterations in the exposed offspring using the same search algorithm in three databases: *PubMed, Web of Science*, and *EBSCO*. The algorithm (Supplementary Table 1) included terms related to hypertensive disorders of pregnancy and neurodevelopment of the offspring; the search was restricted to studies published in Spanish and English from 2000 to 2024. The screening of titles and abstracts as well as the extraction of the information from the selected studies were performed in duplicate; all discrepancies were resolved by consensus. This review was registered in PROSPERO (*International Prospective Register of Systematic Reviews*) before its conduction (registration number, 1067704; registered on March 22, 2024).

### Study question and eligibility criteria

We aimed to answer the question: do hypertensive disorders of pregnancy increase the risk of neurodevelopmental alterations in the exposed offspring? We followed the PECOS statement (population, exposure, comparators, outcomes, and study design) to define the eligibility criteria for the selection of studies [25] (Supplementary Table 2). Were ineligible studies based on children conceived by assisted reproductive technologies; those that considered psychiatric disorders, intellectual disabilities, cerebral palsy, academic performance, or intelligence quotient as their outcomes; and those with chronic hypertension or HELLP syndrome as their exposures of interest. We considered eligible only cohort (prospective or retrospective) and case-control studies whose main exposures were hypertensive disorders that began after 20 weeks of gestation. We excluded cross-sectional designs, systematic reviews, meta-analyses, letters to the editor, commentaries without data, case reports, and publications without peer review.

### Data extraction and quality assessment

The following information was extracted in duplicate (LMZ, LCU) from the studies that were selected in the full-text review: first author, year of publication, country where the study was conducted, study design; sample size, sex of participants, and gestational age; ages of participants; information on the diagnosis of hypertensive disorders of pregnancy and type; determination of neurodevelopmental delay; adjustment covariates used; and the estimated effect measure (risk ratio [RR] and odds ratios [OR]) with their respective confidence intervals (CI). When the selected studies reported linear regression coefficients (β) of the scores on the neurodevelopmental scales, we also recorded them in the extraction table as effect measures.

To assess the quality of the selected studies, we used the Newcastle-Ottawa Scale (NOS) for observational cohort and case-control studies [26]. This scale is composed of eight items grouped into three domains: I) the selection of study groups, II) the comparability of the groups, and III) the measurement of the exposure or results of interest. Each domain can be rated with a maximum score of four, two, and three stars, respectively; therefore, the maximum score of the scale can add up to nine stars. We used the suggested cut-off points to rate the quality of the studies as high quality (≥7 stars), moderate quality (4 to 6 stars), and low quality (≤3 stars). The evaluations were carried out in duplicate and independently (LMZ, EHS); the discrepancies that emerged in the assigned ratings were resolved by consensus between the evaluators.

### Statistical analysis

Quantitative synthesis of the selected articles was performed using meta-analysis to estimate a pooled OR with its respective 95% confidence interval of the association between HDP and delayed neurodevelopment in the offspring. We performed random-effects meta-analyses considering variability of estimates across studies [27]; when comparable data (i.e., OR and RR) were available in three or more studies, we performed separate meta-analyses by neurodevelopment domain (i.e., fine/gross motor, language, and cognition). We assessed the presence of statistical heterogeneity across the selected studies using the Q test and Higgins’ I^2^ statistic, this is expressed as percentages that ranged from 0 to 100% and indicate the total variation due to heterogeneity among studies and not to chance alone [28]. We used the Cochrane criteria to interpret heterogeneity, which was considered non-relevant with an I^2^ < 30%, as moderate with an I^2^ of 30–60%, and as considerable with an I^2^ ≥ 75%. In cases where we identified moderate heterogeneity (I^2^ > 30%), we performed meta-analyses by subgroups (e.g., child’s age at neurodevelopment assessment, neurodevelopmental scale and HDP definition) to identify potential sources of heterogeneity. These analyses, however, were limited because the meta-analysis with moderate heterogeneity included only six to seven publications; thus, some results were reported only for the subgroups with at least three studies to pool. Potential publication bias was assessed using funnel plots to assess asymmetries in conjunction with Egger’s (*p-*value < 0.10*)* and Begg’s (*p-*value < 0.05) tests [29, 30]. Finally, as a sensitivity analysis, we assessed the effect of each individual study on the pooled OR for each meta-analysis by eliminating one study at a time. All statistical analyses were performed using the Stata statistical package (version 15.1, release 2020; StataCorp, College Station, TX, USA).

We rated the certainty of the evidence following the GRADE (Grading of Recommendations Assessment, Development, and Evaluation) methodology; this tool considers the study design, risk of bias, inconsistency, indirectness, imprecision, and publication bias as relevant factors to assess the certainty of evidence of each meta-analysis [31].

## Results

**Fig. 1:**
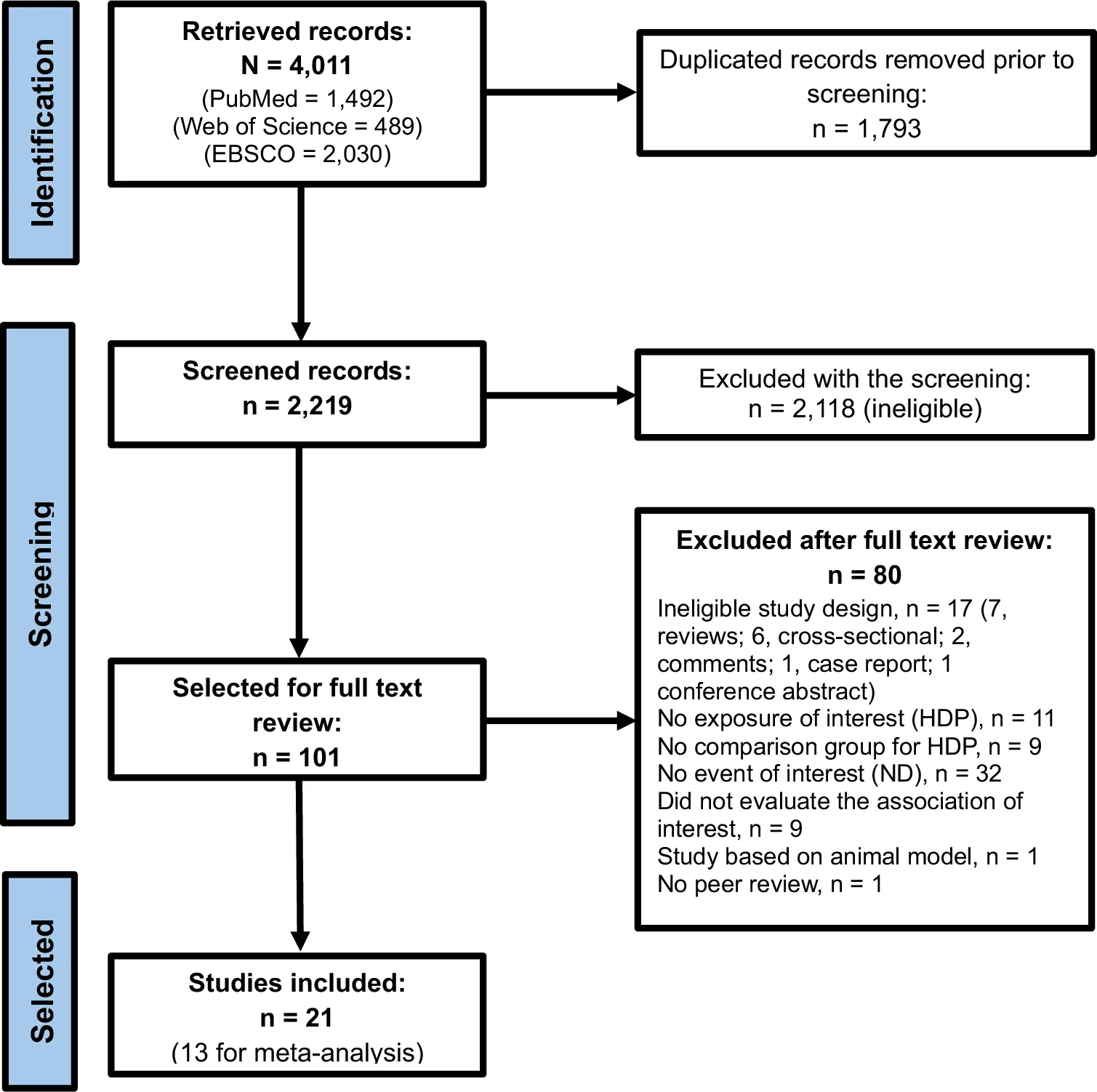
Flowchart of the selection process of studies evaluating the association between hypertensive disorders of pregnancy and delayed neurodevelopment in the offspring

We identified 4011 original articles from the three databases included in the review (PubMed, Web of Science, EBSCO) and discarded 1793 duplicates. We reviewed the titles and abstracts of 2219, leaving 101 for full-text review (Fig. 1). Finally, 80 articles were excluded, and 21 were selected for data extraction; only 13 had data to include in the meta-analysis. The main reasons for exclusion were that they did not evaluate the event of interest (*n* = 32), the study design was not eligible (*n* = 17), they did not assess HDP (*n* = 11), they did not evaluate the association of interest (*n* = 9), or they did not have a comparison group (*n* = 9).

### Qualitative synthesis

Characteristics and data extracted from the 21 articles are summarized in Table 1. Only one study was a case-control study, and 20 were cohort studies, 15 of which were prospective and five retrospective; most were conducted in Asian countries (*n* = 10), the others were from America (*n* = 5), Europe (*n* = 3), and Oceania (*n* = 3). The sample size of the studies ranged from 60 to 1,972,861; the age ranged from 6 months to 10 years; 15 included boys and girls, and six did not report this data. Most included premature and low birth weight infants (*n* = 14) in their samples; three included preterm infants without reporting birth weight [32–34]; three included full-term infants [19, 35, 36], only one of these with normal birth weight [36]; and one did not report related information [16]. The most frequently assessed prenatal exposure in relation to neurological development was preeclampsia (*n* = 10), and the remaining included HDP (*n* = 11) with variations in definitions (i.e., combinations of GHTN, PE, eclampsia, and pre-pregnancy HTN). The most used neurodevelopment screening scales were Bayley (versions II and III, *n* = 7) and the ASQ (Ages & Stages Questionnaire, *n* = 2); one-third used other scales (*e.g*., Gessell, Kaufman, Griffiths), and two were based on medical records [37, 38]. Most studies reported some association (*n* = 16), either crude or adjusted for maternal and child-related confounders such as sex, age, breastfeeding, and gestational age at birth (Table 1).

**Table 1:** Studies that assessed the association between hypertensive disorders of pregnancy and offspring’s neurodevelopmental delay

Author & year Country	Study design	n (n by sex) GA/BW	Age (years or months)	Ascertainment of HDP	Ascertainment of ND delay	Confounders considered	Measure of effect (IC95%)
Huang et al., 2023. China [36].	Prospective cohort.	2576 (♀, 1209; ♂, 1367) GA: mean ± SD, 39.44 ± 1.34 wk. BW: mean ± SE, 3387 g ± 446.95.	1 year.	HDP extracted from medical records: chronic HTN/PE w/ superimposed chronic HTN; GHTN/PE confirmed w/ACOG criteria.	Suboptimal ND based on Bayley-III scale in the following domains: cognitive, receptive communication, expressive communication, gross and fine motor skills. Suboptimal ND included emerging and at-risk domains.	Maternal: age, education, pre-pregnancy BMI, parity, type of conception (spontaneous or using ART), and study center Children: sex, breastfeeding duration, and age at examination.	**GHTN/PE:** Cognition: aβ = –0.18 (–0.54, 0.17); expressive communication: aβ = –0.17 (–0.52, 0.19); fine motor: aβ = 0.02 (–0.26, 0.31); gross motor: aβ = –0.21 (–0.52, 0.10). Cognition: aRR=1.34 (0.83, 2.16); expressive communication: aRR=0.76 (0.28, 2.10); fine motor: aRR=0.86 (0.27, 2.77); gross motor: aRR=1.20 (0.67, 2.12).
Chang et al., 2023. Taiwan [17].	Retrospective cohort.	482 (♂ 241; ♀ 241). Preterm infants & BW: ≤1500 g.	2 years.	PE w/severe features extracted from medical records: SBP ≥ 160 or DBP ≥ 110 mmHg w/signs of organ dysfunction.	ND alteration based on the Bayley-III scale in 3 domains: cognitive, language, and motor skills. ND alteration defined as a score <85 (<1 SD) in any domain.	Maternal age, GA, parity, DM, GDM, mode of delivery, placenta previa, placental abruption, chorioamnionitis, SES, antenatal corticosteroids, & abnormal FHR.	Cognition: aOR= 4.38 (1.50, 12.71); language: aOR= 4.15 (1.99, 8.64); motor: aOR= 1.43 (0.71, 2.90).
Kim et al., 2022 South Korea [37].	Retrospective population-based cohort.	1,972,861 ♀♂ Included preterm infants & w/low BW.	~6–10 years.	Dx. from medical records (ICD): **GHTN** (O13); **PE/E** (O14.0-O14.2, O14.9, O15.0–O15.2, O15.9).	Medical records (ICD): Speech and language impairment (ICD-10: F80.0–F80.2, F80.8, F80.9, F98.5).	NR	**GHTN:** aOR= 1.19 (0.98, 1.44). **PE/E:** aOR= 1.34 (1.19, 1.50).
Palatnik et al., 2022. USA [19].	Prospective cohort.	1179 (♀, 570; ♂, 609). Term infants, ≥37 wk.	12 & 24 months.	**HDP:** GHTN & PE PE: SBP ≥ 140 or DBP ≥ 90 mmHg measured twice, 4 h apart + proteinuria ≥300/24 h or 2+ test strips on ≥2 occasions.	Bayley’s -III scores at 12 & 24 months: cognitive, motor, & language. Cut-off points: <85%.	Parity, baseline maternal age, maternal BMI, assigned treatment group, & baseline thyroid.	**12 months:** Cognitive: aOR=0.81 (0.34, 1.94); motor: aOR=0.50 (0.18, 1.40); language: aOR= 0.92 (0.49-1.71). **24 months:** Cognitive: aOR= 0.79 (0.44, 1.42); motor: aOR= 0.70 (0.29-1.68); language: aOR= 2.01 (1.24, 3.25).
Koparkar et al., 2022. India [55].	Prospective cohort.	313 (♂, 179; ♀, 134). Included preterm/ term & low BW infants.	5-7 years.	**Dx. of PE:** SBP ≥ 140 &/or DBP ≥ 90 mmHg w/proteinuria 1+ from test strips.	Cognitive function: Kaufman battery including Atlantis, word order, and reasoning patterns; verbal fluency, Kohs block design & Coding tests (Wechsler intelligence Scale III for children).	Child’s sex & age, family’s SES, & mother’s education.	*RR/OR unavailable*. Atlantis: aβ = −0.23 ( −0.49, 0.03). Word order: aβ = −0.13 ( −0.38, 0.11). Pattern recognition: aβ = −0.092 ( 0.33, 0.14). Verbal fluency: aβ = 0.15 ( −0.10, 0.40). Coding A: aβ = −0.21 (−0.47, 0.06). Kohs block design: aOR= 0.57 (0.34, 0.96).
Sala et al., 2022. Argentina [33].	Prospective cohort.	132 (♂ 73; ♀ 59). Includes preterm infants of 36 wk.	6 months.	**HDP:** GHTN, PE & E. GHTN: SBP ≥ 140 or DBP ≥ 90 mmHg after 20 wk. of GA. PE: SBP ≥ 140 or DBP ≥ 9 w/persistent proteinuria >0.3 g/L in 24 h. E: PE + convulsions.	At risk of ND delay or ND delay based on the Bayley-III scale in 3 domains: cognitive, language (receptive/expressive communication), & motor (fine/gross). Cut-off point to define at risk of ND delay or ND delay: score <85 points.	Number of children & exclusive breastfeeding at 6 months.	General: aOR= 3.87 (1.26, 13.83). Language: aOR= 6.08 (1.15, 57.05). Cognitive: aOR= 2.82 (0.52, 23.76). Motor: aOR= 3.01 (0.98, 10.64).
Manovitch et al., 2022. Israel [32].	Retrospective cohort [*published as case-control*].	71 (♂♀). Preterm infant, <32 wk.	6, 12 & 24 months.	**PE dx.:** BP > 140/ ≥ 90 mmHg w/proteinuria 300 mg/24 h or ≥1+ in test strips.	GMDS: motor skills, personal-social interaction, language ability, hearing, eye-hand coordination, & performance skills.	Those exposed to PE were matched by GA, sex, & to those unexposed to PE.	*RR/OR unavailable*. Children exposed to PE had higher scores on the Griffiths scale compared to unexposed (NS). No measure of effect was reported.
Scime et al., 2021. Canada [34].	Prospective cohort.	1554 (♂, 825; ♀, 729). Includes preterm & term infants (GA, mean ± SD: 39.0 ± 1.7wk).	3 years.	**Dx. of HDP** from medical records (ICD): includes chronic HTN, GHTN, PE, & E. Chronic HTN: SBP ≥ 140 mmHg & DBP ≥ 90 mmHg w/onset prior to pregnancy (O10). GHTN: new onset >20wk (O13). **PE:** HTN w/proteinuria (O14). **E:** (O15).	Five domains of ASQ-3: communication, problem-solving, personal-social (cognitive development), gross motor, & fine motor (motor development). ASQ-3 cutoffs: Any delay ≤1 SD below the mean in at least 1/5 domains; cognitive delay, ≤1 SD below the mean in at least 1/3 cognitive domain; motor delay, ≤1 SD below the mean in at least 1/2 motor domain.	Sociodemographic vulnerability, maternal age, pre-pregnancy overweight/obesity, prenatal depression, & child’s sex.	**HDP:** Any delay, aRR= 1.21 (0.94, 1.55); Motor delay, aRR= 1.20 (0.89, 1.63); Cognitive delay, aRR= 1.28 (0.86, 1.89). **PE/E:** Any delay, aRR = 1.29 (1.00, 1.65); Motor delay, aRR= 1.29 (0.94, 1.76); Cognitive delay, aRR= 1.32 (0.88, 1.99).
Noda et al., 2021. Japan [38].	Population-based retrospective cohort.	43,854 (♂, 22,439; ♀, 21,415). GA: ≤37wk. BW: ≤2500 g.	3 years.	**HDP** extracted from medical records: GHTN, PE, superimposed PE or E.	Problems with motor (gross/fine) & mental development (cognition/behavior). Cases were those w/medical indications of: 1) required detailed check-ups, hospital examination, 2) required medical therapy, or 3) under treatment.	Maternal age, multiple pregnancy, parity, maternal smoking & alcohol consumption, & child’s sex.	**Motor problem:** cOR= 1.80 (1.13, 2.87) aOR= 1.69 (1.06, 2.70) **Mental problem:** cOR= 2.01 (1.36, 2.97) aOR= 1.79 (1.21, 2.66)
Bandiya et al., 2019. India [56].	Prospective cohort. [*published as case-control*].	60 (♂, 34; ♀, 26). Preterm infants 34- < 37 wk. Include low BW infants.	40wk (10 months)	**Physician’s dx. of PE** according to the ACOG criteria.	Neurobehavioral score using the Neurobehavioral Assessment for Premature Infants (NAPI): motor development and vigor & alertness orientation.	None.	*RR/OR unavailable*. Means ± SD Motor development and vigor: without PE: 70.0 ± 11.48 vs. w/PE: 60.1 ± 9.56. Alertness orientation: without PE: 45.6 ± 13.33) vs. w/PE: 37.45 ± 11.04.
Adane et al., 2018. Australia [16].	Prospective cohort.	771 (♂; ♀). 708 (♂; ♀). NR	1-66 months (Median, 5 years).	**Maternal self-reported HDP diagnosis (including pre-existing HTN)**. [During the study, the Australian & New Zealand’s guidelines for the management of HDP defined PE as SBP or DBP ≥ 140/90 mmHg w/proteinuria ≥300 mg/24-h or maternal dysfunction of other organs].	Gross motor developmental delay using ASQ. Gross & fine motor skills; language, cognitive, & communication skills; & general knowledge obtained from the Australian Early Development Census (AEDC): ≤25 percentile was defined as vulnerable & at-risk.	Maternal age, residence area, parity, education, smoking, physical activity before delivery, & pre-pregnancy BMI.	**Gross motor development delay (ASQ):** aRR= 0.89 (0.37, 2.13) **AEDC** Gross & fine motor skills: aRR= 0.88 (0.54, 1.44); Language & cognitive skills: aRR=0.92 (0.54, 1.57); Communication skills & general knowledge: aRR= 0.88 (0.56, 1.35).
Girchenko et al., 2018. Finland [35].	Prospective cohort.	2504 (♂, 1267; ♀, 1237). Only term infants: ≥37 wk.	Mean, 42.1 months (range: 23.2–68.8).	Extracted from the Medical Birth Registry & verified by a panel. **GHTN:** BP ≥ 140/90 mmHg on 2+ occasions w/at least 4 h of difference. **PE:** BP ≥ 140/90 mmHg on 2+ occasions w/at least 4 h of difference w/proteinuria ≥300 mg/24 h. Both diagnoses among women w/normal BP before 20wk. of GA.	Delay in developmental milestones using the ASQ-3 scale, domains: communication, gross & fine motor skills, problem-solving, & personal/social skills. Delay in a specific domain was defined as ≤2 SD in the scores for age.	Maternal: age at delivery, type of delivery, tobacco & alcohol use during pregnancy, & education. Child: GA, BW, sex, & age at follow-up.	**GHTN** Communication: aOR= 1.45 (0.56, 3.74); Fine motor: aOR= 1.76 (0.80, –3.88); Gross motor: aOR= 0.75 (0.27, 2.11); Problem solving: aOR= 1.82 (0.84, 3.94); Social skills: aOR= 1.09 (0.38, 3.13). **PE** Communication: aOR= 2.47 (1.10, 5.59); Fine motor: aOR= 0.51 (0.15, 1.69); Gross motor: aOR=1.82 (0.91, 3.62); Problem solving: aOR= 1.74 (0.81, 3.71); Social skills: aOR= 1.09 (0.38, 3.18). **PE & severe delay** (excluding those w/pregnancy problems such as obesity & diabetes): aOR= 2.19 (1.30, 3.70) with problems of pregnancy such as obesity and diabetes): aOR= 2.19 (1.30, 3.70).
He et al., 2017. China [41].	Case-control.	1337 (♀, 798; ♂, 539). Preterm infants: 26–36wk. BW: 650– 2350 g.	18 months.	PE/pregnancy-induced HT.	Abnormal neurodevelopment using Gesell’s developmental schedules: adaptive parameters, gross & fine motor skills, language comprehension & expression, & personal social skills.	Maternal age & education, GDM, meconium, GA, BW, multiple birth, & type of delivery. Cases were matched by age, sex, parental educational level, & occupation to controls.	aOR= 54.4 (12.44, 237.88).
Bharadwaj et al., 2018. India [57].	Prospective cohort.	143 (♂, 76; ♀, 67). Preterm infants: 34– <37 wk. Include low BW.	1 year.	**PE by medical dx.:** SBP ≥ 140 or DBP ≥ 90 mmHg at least 2 times 4 h apart after 20wk of GA in a woman w/BP previously normal & proteinuria >300 mg/24 h urine or a dipstick w/1+ or protein/creatinine ratio >0.3.	Developmental Assessment Scale for Indian Infants (DASII): motor, cognitive, linguistic & social personal development. Motor or mental developmental delay defined as a quotient <70.	None.	*RR/OR unavailable*. Motor quotient: PE 97.6 (91.4, 101.1) Mental quotient: PE 100 (94.8, 102.5)
Warshafsky et al., 2016. Canada [58].	Prospective cohort.	235 (♂, 131; ♀, 104). (1 year, 197; 2 years, 170; 3 years, 99). Includes infants <37 & ≥37wk. Includes infants w/ IUGR.	1, 2 & 3 years.	**Severe PE at hospital admission:** BP > 140/90 mmHg & proteinuria >300 mg/24 h or ≥1+ repeated in test strips. Plus 1+ of: SBP ≥ 160, DBP ≥ 110, proteinuria ≥5 g/24 h or ≥3+ in test strips, oliguria (≤500 mL/24 h), brain or visual changes, epigastric pain, thrombocytopenia (<150,000 × 109/L), AST > 46 U/L & ALT > 40 U/L, serum creatinine >106 μmol/L, pulmonary edema or cyanosis, IUGR, or E.	Neurodevelopmental failure based on ASQ: gross & fine motor skills, communication, personal social skills, & problem-solving.	Maternal use of MgSO4, smoking, SES, parity, & breastfeeding. Child’s sex, GA, IUGR. Women w/PE were matched to those normotensive without a prior history of PE, parity, maternal age, & race.	1 year: aOR= 0.90 (0.24, 3.34); 2 years: aOR= 0.63 (0.19, 2.09); 3 years: aOR= 2.31 (0.63, 8.53).
Bolisetty et al., 2014. Australia [59].	Retrospective cohort.	1472 (♂, 1215; ♀, 257). Preterm infants: 23–28wk. (mean, 27 wk) with & w/o IVH. Mean BW, 945 g (IQR: 776–1,110).	2-3 years.	**Pregnancy-induced hypertension (PIH)** obtained from medical records.	Moderate to severe neurosensory disability defined as ND delay based on the GMDS-GQ & the BSID-II, MDI. Neurosensory disability defined w/score ≥-2SD in GMDS-GQ or BSID-II, MDI.	Child’s sex, chronic lung disease, SGA, sepsis, & severe retinopathy of prematurity.	aβ (SE) = 0.203 (0.22). aOR= 1.22 (0.79- 1.88).
Kerstjens et al., 2013. Holland [39].	Prospective cohort.	834 (♂; ♀). Preterm infants: 32- < 36 wk. Includes infants w/IUGR.	4 years.	**HDP** dx. from medical records, including HELLP syndrome or PE/E.	Developmental delay using the Dutch version of the ASQ: communication, fine & gross motor skills, problem-solving ability, & personal social functioning. Developmental delay was defined as: ASQ <−2.00 SD.	None. *[OR estimated from raw data]*.	cOR= 1.23 (0.67, 2.28).
Leitner et al., 2012. Israel [60].	Prospective cohort.	120 (♂; ♀). Infants w/IUGR. Includes preterm, full-term & low BW infants.	9-10 years.	**Self-reported PE & GHTN**. PE: SBP ≥ 140 or DBP> 90 mmHg after 20wk of GA in women with prior normal BP & proteinuria (0.3+ g/24 h or 2+ in test strips). GHTN: SBP ≥ 140 or DBP ≥ 90 mm Hg after 20 wk of GA w/o proteinuria. PE & GHTN followed the ACOG criteria.	Academic achievement using the Kauffman Assessment Battery for Children (K-ABC).	None.	*RR/OR unavailable*. Mean ± SD **ND:** Maternal normotension: 87.47 ± 8.24; GHTN: 85.48 ± 9.12; PE: 84.29 ± 12.30. **Kaufman:** Maternal normotension: 602.90 ± 80.38; GHTN: 598.16 ± 79.05; PE: 612.94 ± 98.89.
Whitehouse et al., 2012. Australia [61].	Retrospective cohort.	1389 (♂, 714; ♀, 675). Includes preterm & full-term infants. Includes infants w/low & normal BW.	10 years.	Dx. from medical records. **HDP:** GHTN & PE. **GHTN:** SBP ≥ 140 or DBP ≥ 90 mmHg in women w/normal BP <24 wk GA. **PE:** GHTN + proteinuria 300 mg/24 h.	Verbal ability with the Peabody picture vocabulary test-revised (PPVT-R) as a measure of receptive vocabulary (verbal IQ with mean = 100 & SD = 15).	Maternal: age at conception, education, household income, tobacco & alcohol consumption in pregnancy, essential HTN, use of antihypertensives, spontaneous onset of labor, & parity. Children: GA, BW, Apgar score at 5 min., sex, McMaster family assessment device score at 3 & 5 years.	**GHTN/PE:** PPVT-R, aβ = -1.83 (-3.48, -0.17). **GHTN:** PPVT-R, aβ = -1.71 (-3.39, -0.03). **PE:** PPVT-R, aβ = 3.53 (-8.41, 1.35).
Spinillo et al., 2009. Italy [42].	Prospective cohort.	754 (♂, 394; ♀, 360). GA: 24–33 wk (mean ± SD, 30.4 ± 2.4). BW: ≤1500 g (mean ± SD: 1441 g ± 465).	2 years.	**PE** from medical records, undefined criteria.	General neurodevelopmental alteration using the BSID-II, MDI ≤ 84.	Maternal: age, social class, tobacco, education, nulliparous, PPD, placenta previa, prenatal steroid use, PPROM, spontaneous onset of labor, use of tocolytics, & delivery by C-section. Children: GA, BW, % of expected BW at birth, sex, non-reassuring FHR, pH≤7.2 in the umbilical artery, & SGA.	*RR/OR unavailable*. BSID-II, MDI Mean ± SD: Normal: 156 ± 26.4; moderate impairment: 24 ± 22.7; moderate to severe impairment: 4 ± 7.5. Moderate-severe ND: ♀ & ♂: cOR= 0.60 (0.37, 0.95). **General alteration:** ♀ & ♂: aOR= 0.52 (0.32, 0.85); ♂ w/PE: aOR=5.6 (2.0, 15.4) vs. ♀ w/PE; ♂ w/o/PE: aOR=1.4 (0.92, 2.01) vs. ♀ w/o/PE.
Silveira et al., 2007. Brazil [40].	Prospective cohort.	86 (♂; ♀). Includes preterm & infants w/BW < 1500 g.	12 y 18 months	**PE dx.:** SBP ≥ 140 or DBP ≥ 90 mmHg w/onset after 20wk of GA w/proteinuria >300 mg/24 h in the absence of HTN & prior renal disease.	Neurodevelopmental delay using the BSDI-II, MDI & PDI. Moderate delay: score below 85 (-1SD) in the MDI or PDI. Severe delay: score below 70 (-2SD) in the MDI or PDI.	None. *[OR estimated from raw data]*.	**12 months:** Mean ± SD: MDI, PE: 79.21 ± 3.14; w/o PE: 80.09 ± 0.9. cOR: 2.85 (0.47, 30.22). Mean ± SD: PDI, PE: 80.29 ± 3.52; w/o PE: 79.73 ± 1.19. cOR: 2.85 (0.47, 30.22). **18 months:** Mean ± SD: MDI, PE: 82.71 ± 0.81; w/o PE: 82.45 ± 0.51. cOR: 1.25 (0.30, 5.5). Mean ± SD: PDI, PE: 83.29 ± 1.17; w/o PE: 83.8 ± 0.8. cOR: 0.65 (0.25, 1.73).

Symbols: girls, ♀; boys, ♂

*ACOG* American College of Obstetricians and Gynecologists, *AEDC* Australian early development census, *ALT* alanine aminotransferase, *ASQ* ages, and stages questionnaire, *AST* aspartate aminotransferase, *aOR* adjusted odds ratio, *aRR* adjusted risk ratio, *ART* assisted reproductive technology, *BW* birthweight, *BMI* body mass index, *BSID-II* *MDI*, Bayley scales of infant development-second edition, mental development index, *BSID-II PDI* Bayley scales of infant development-second edition, psychomotor development index, *CI* confidence interval, *DASII* developmental assessment scale for Indian infants, *BP* blood pressure, *DBP* diastolic blood pressure, *DM* diabetes mellitus, *dx* diagnosis, *E* eclampsia, *FHR* fetal heart rate, *g* grams, *g/L* grams/liter, *GA* gestational age, *GDM* gestational diabetes mellitus, *GHTN* gestational hypertension, *GMDS-GQ* Griffiths general mental development quotient scale, *h* hours, *HDP* hypertensive disorders of pregnancy, *HELLP* hemolysis, elevated liver enzymes, and low platelets, *HTN* hypertension, *ICD* international classification of diseases, *IVH* intraventricular hemorrhage, *IQ* intellectual quotient, *IQR* interquartile range, *IUGR* intrauterine growth restriction, *K-ABC* Kauffman assessment battery for children, *NAPI* neurobehavioral assessment for premature infants, *ND* neurodevelopment, *NR* not reported, *NS* not statistically significant, *MDI* mental development index, *mg* milligrams, *mL* milliliters, *mmHg* millimeters of mercury, *MgSO4* magnesium sulfate, *OR* odds ratio, *µmol/L* micromoles per liter, *PE* preeclampsia, *PMR* premature rupture of membranes, *PPD* premature placental abruption, PPROM preterm premature rupture of membranes, *PPVT-R* Peabody picture vocabulary test-revised, *PIH* pregnancy-induced hypertension, *RR* risk ratio, *SBP* systolic blood pressure, *SD* standard deviation, *SE* standard error, *SES* Socio economic status, *SGA* small for gestational age, *U/L* units per liter, *vs* versus, *w* with, *wk* weeks *w/o* without

### Quality assessment

Most studies (*n* = 18) were rated as high quality (≥7 stars) according to the Newcastle-Ottawa scale for cohort studies, while the only case-control study (5 stars) was of moderate quality. Two other cohort studies (5–6 stars), originally published as case-control studies, were also of moderate quality (Supplementary Table 3).

### Quantitative synthesis

Meta-analyses were based on 13 studies reporting associations; we estimated crude ORs from raw published data in two cases [39, 40]. Identification of potential sources of heterogeneity was restricted by the limited number of studies (9 to 6) to conduct stratified meta-analyses, except for age of the participants.

**Fig. 2:**
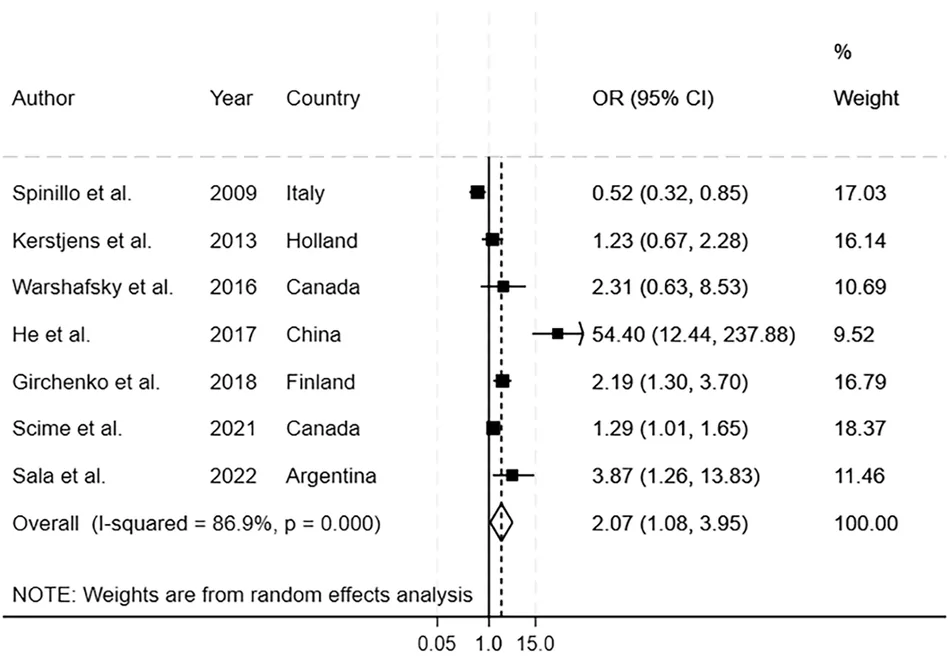
Meta-analysis of hypertensive disorders of pregnancy and delayed global neurodevelopment in the offspring

Based on seven studies with 7214 participants, we found that HDP doubled the risk of delayed global neurodevelopment in the offspring (OR = 2.07; 95%CI: 1.08, 3.95), however, heterogeneity between studies was considerable (I^2^ = 86.9%) (Fig. 2). The effect of HDP on delayed neurodevelopment tended to be greater in studies with participants aged ≤24 months (OR = 4.54; 95%CI: 0.31, 62.39; *n* = 3) and was moderate when participants were older than 24 months (OR = 1.48; 95%CI: 1.11, 1.97; *n* = 4); however, heterogeneity decreased (I^2^ = 24.4%) only for the strata with participants older than 24 months (Supplementary Table 4). A somewhat stronger association was observed for the subgroup of 5/7studies that did not include severe preeclampsia in their definition of HDP (OR = 2.42; 95%CI: 1.02, 5.71), but its heterogeneity increased (I^2^ = 91.1%) and remained considerable (Supplementary Table 5). However, heterogeneity decreased and was not relevant (I^2^ = 24.4%) for the 4/7 studies that used the ASQ to assess global neurodevelopment, although the pooled estimate (OR = 1.48; 95%CI: 1.11, 1.97) was substantially attenuated.

**Fig. 3:**
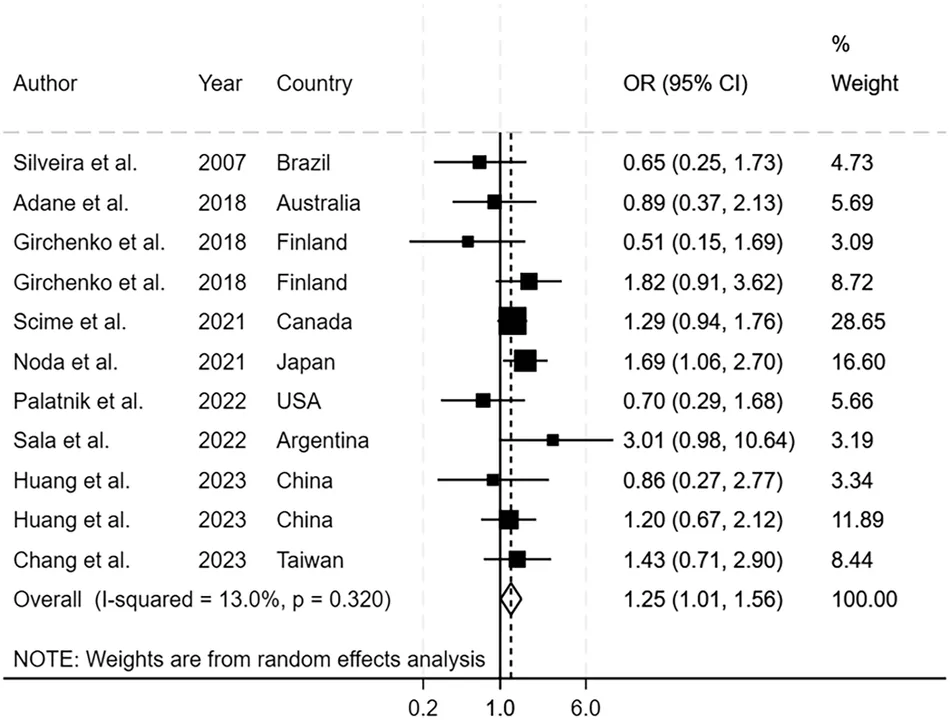
Meta-analysis of hypertensive disorders of pregnancy and delayed motor development in offspring

The motor domain of neurodevelopment was assessed in nine studies with 53,138 participants, most of which considered the combination of fine and gross motor skills; two reported fine and gross motor skills separately [35, 36], and one reported only gross motor skills [16]. Exposure to HDP moderately increased the risk of delayed motor skill development of offspring (OR = 1.25; 95% CI: 1.01, 1.56), with non-relevant heterogeneity (I^2^ = 13.0%) (Fig. 3). The results remained in the same direction (OR = 1.29; 95% CI: 0.87, 1.92) when limiting the meta-analysis to the three studies that assessed gross motor development separately (Supplementary Fig. 1), although no apparent heterogeneity (I^2^ = 0.0%) emerged, the small number of studies limits the statistical power of this test. However, non-relevant heterogeneity was also observed for the main meta-analysis based on nine studies.

**Fig. 4:**
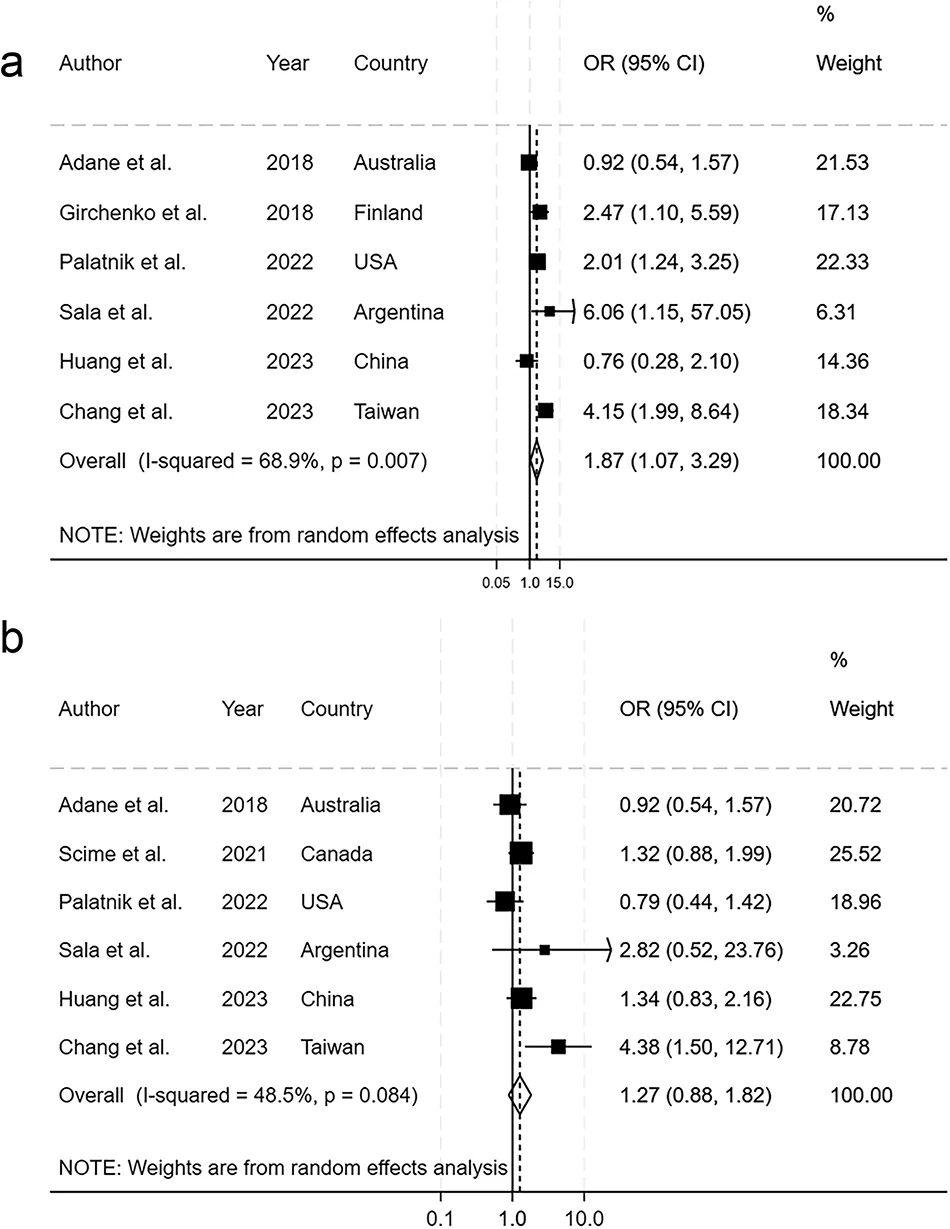
Meta-analysis of hypertensive disorders of pregnancy in relation to delayed language and cognitive development in the offspring. **a** Delayed language development. **b** Delayed cognitive development

Language development was assessed in six studies with 7581 participants (Fig. 4a). Exposure to HDP was also associated with an increased risk of delayed language development (OR = 1.87; 95%CI: 1.07, 3.29) with considerable heterogeneity (I^2^ = 68.9%). Only two studies included participants older than 24 months, so we only performed meta-analyses based on four studies with participants aged ≤24 months, the estimate was similar in direction and magnitude (OR = 2.22; 95%CI: 1.07, 4.61), but heterogeneity decreased little (I^2^ = 63.9%) (Supplementary Table 4). The pooled OR was attenuated in the subgroup (*n* = 5/6) that did not consider severe preeclampsia as part of their definition of HDP (OR = 1.55; 95%CI: 0.90, 2.67) and was stronger in the subgroup (*n* = 4/6) that used the Bayley-III scale (OR = 2.23; 95%: 1.08, 4.62) to assess language development (Supplementary Table 5). Similarly, the heterogeneity decreased little but remained considerable for the subgroup that excluded severe preeclampsia from the HDP definition (I^2^ = 59.2%) and the subgroup that used the Bayley-III scale (I^2^ = 63.9%).

Based on 6 studies with 6631 participants we observed an association between HDP and delayed cognitive development (OR = 1.27; 95%CI: 0.88, 1.82), but it did not reach statistical significance, and heterogeneity (I^2^ = 45.8%) was moderate (Fig. 4b). Similarly, the association remained when we limited the meta-analysis to the four studies that included participants aged ≤24 months (OR = 1.56; 95%CI: 0.79, 3.08); however, heterogeneity increased (I^2^ = 64.2%) indicating unaccounted sources of heterogeneity (Supplementary Table 4). We observed a weaker association when the meta-analysis was limited to the 5/6 studies that did not consider severe preeclampsia in their definition (OR = 1.14: 95%CI: 0.90, 1.45), interestingly, no heterogeneity (I^2^ = 0.0%) was observed within this subgroup (Supplementary Table 5). Stronger associations emerged when we limited the meta-analysis to the 4/6 studies that used the Bayley-III scales to assess cognitive development (OR = 1.56; 95%CI: 0.79, 3.08); however, the heterogeneity (I^2^ = 64.2%) increased and was considerable rather than moderate.

### Sensitivity analysis

In the sensitivity analysis removing one study at a time from the meta-analysis of delayed global neurodevelopment (Supplementary Table 6), we observed that the exclusion of the study by He et al. [41]. caused the greatest attenuation of the OR (OR: 2.07 vs. 1.38) and of heterogeneity (I^2^: 86.9% vs. 76.3%), while the exclusion of the study by Spinillo et al. [42] caused the greatest increase in the OR (OR: 2.07 vs. 2.68). The study by He et al. [41] is the only case-control design that also reported the strongest association (OR = 54.4) with wide confidence intervals (indicating greater imprecision in the estimate), while the study by Spinillo et al. [42] is the only one that reported a statistically significant reduction in risk (OR = 0.52) instead of an increase due to HDP; both studies included preterm and low birth weight participants.

Consistent with what we observed in the meta-analysis of delayed motor development (Fig. 3), the exclusion of one study at a time (Supplementary Table 7) had little effect on the observed heterogeneity, and it remained non-relevant (I^2^ < 30%). Whereas the OR was attenuated (OR: 1.25 vs. 1.18) or strengthened (OR: 1.25 vs. 1.30) with these exclusions, the changes in OR were less than 6%.

On the other hand, in the meta-analysis evaluating delayed language development (Supplementary Table 8), the exclusion of the study by Adane et al. [16] showed the strongest association (OR: 1.87 vs. 2.26), while the exclusion of Chang et al. [17] showed the weakest association (OR: 1.87 vs. 1.54); both exclusions reduced the heterogeneity from considerable to moderate (I^2^ of 52.3% and 59.2%, respectively).

Finally, excluding the study by Adane et al. [16], Palatnik et al. [19] and Chang et al. [17] from the meta-analysis assessing delayed cognitive development (Supplementary Table 9) showed similar changes in the associations (-10.2% and +10.2%), making the associations stronger (OR: 1.27 vs. 1.40) or weaker (OR: 1.27vs. 1.14). However, excluding Chang et al. [17] eliminated the observed heterogeneity (I^2^: 48.5% vs. 0.0%). Particularly, this study [17], included in the meta-analyses of delayed language and cognitive development, was the only one that included preterm and very low birth weight participants.

### Publication bias assessment

Funnel plots of the four meta-analyses with their respective Egger’s and Begg’s tests are shown in the supplementary material. Overall, no relevant asymmetries were observed in the meta-analyses of delayed global neurodevelopment (Supplementary Fig. 2A) or delayed motor (Supplementary Fig. 2B), language (Supplementary Fig. 2C), and cognitive (Supplementary Fig. 2D) development. Consistent with the funnel plots, the Egger’s and Begg’s tests did not suggest possible publication bias (Egger’s *p* ≥ 0.22 and Beeg’s *p* ≥ 0.12). Still, these results should be interpreted with caution, especially those assessing delayed language and cognitive development as outcomes, as they were based on only six studies.

The certainty of the evidence according to the GRADE assessment suggests moderate evidence for the association of HDP with delayed global neurodevelopment and delayed motor and language development; however, the evidence was rated low for delayed cognitive development (Supplementary Tables 10–13).

## Discussion

Our results suggest that *in utero* exposure to hypertensive disorders of pregnancy is associated with an increased risk of delayed neurodevelopment in the offspring, specifically apparent in the motor and language domains. Although the weakest association of HDP was observed with delayed motor development (OR = 1.25), the heterogeneity was not relevant, whereas the strongest associations appeared with delayed global (OR = 2.07) and language development (OR = 1.87), both with considerable heterogeneity between studies. Nonetheless, the certainty of the evidence was moderate according to the GRADE assessment, and there was no evidence of publication bias. We also observed a small but not statistically significant increased risk of children’s cognitive delay in relation to HDP (OR = 1.27).

An association between HDP and offspring’s global neurodevelopmental delay (OR = 1.77; 95%CI: 1.21, 2.59) was reported in a recent meta-analysis that included 32 publications [43]. Our estimate (OR = 2.07) is comparable in magnitude, but the heterogeneity across these 32 studies was much higher (I^2^: 99.58%) than what we observed (I^2^: 86.9%), which could be due to the inclusion of cross-sectional studies unlike the present. Still, a relatively recent narrative review concluded that there is no conclusive evidence [44]. Our results, although suggestive, are not conclusive since most studies did not account for common causes of HDP and offspring’s neurodevelopment, such as preterm delivery and its consequences.

While the mechanism that explains how HDP could adversely affect the offspring’s neurodevelopment is not well understood, the pathogenesis of PE, such as angiogenic imbalance, utero-placental hypoperfusion and ischemia, systemic maternal inflammation, and oxidative stress, might be implicated [4]. Fetal hypoxia caused by placental dysfunction might also disturb the development of the human fetus’s brain [45]; hypoxia has also been associated with alterations in the early development of animal models and in their behavior [46]. Alterations in human fetal brain structures and abnormal functional connectivity have also been reported among children born from preeclamptic pregnancies [45, 47], further supporting a plausible association.

Limitations of the present study includes the considerable heterogeneity between studies of the pooled estimate for delayed global neurodevelopment and delayed language development (I^2^ > 60%). In the meta-analysis of global neurodevelopment, heterogeneity was smaller (I^2^ < 24.4%) for the studies with participants older than 24 months but was greater when the participants were ≤24 months. Similar changes in heterogeneity were observed for delayed language and cognitive development when the meta-analyses were limited to participants aged ≤24 months. Additionally, our results suggest that the definition of HDP and the type of neurodevelopmental scales used across studies are important sources of heterogeneity that could account for the inconsistency of the prior studies. Unfortunately, we were unable to identify other sources of heterogeneity given the small number of studies for these meta-analysis, thus subgroup meta-analyses were also limited.

The use of several neurodevelopmental scales, which were applied at varying ages, may explain some of the observed heterogeneity because they could have limited comparability across ages. In the present, seven out of 21 studies used the Bayley scale (II or III), whereas the remaining used other developmental scales, which may have contributed to the considerable heterogeneity. Even though such tools are valid to assess the neurodevelopment of children, they tend to assess specific domains such as cognition, motor, and language development at varying ages; thus, they are focused on different milestones [48–51]. In addition, these scales are simple screening tools that are accessible and easy to apply, but they have limited utility as diagnostic tools and are not widely implemented as part of the routine care of the pediatric population to allow a timely identification of children at a higher risk of neurodevelopmental delay [52].

Furthermore, several studies included premature and low birthweight infants in their samples; thus, we were unable to assess the independent effect of HDP on neurodevelopment. A recent meta-analysis of global neurodevelopmental delay in relation to HDP reported weaker associations across studies that adjusted for gestational age at birth (OR = 1.16; 95%CI: 0.85, 1.60; *n* = 4) and birthweight (OR = 1.16; 95%CI: 0.79, 1.69; *n* = 3) [43]. We were unable to conduct similar analyses given the limited number of studies included in the present; this important limitation also suggests that our results should be interpreted with caution. Preeclampsia is one of the leading causes of preterm birth [53, 54], however, with the present meta-analysis, it is unclear whether HDP could impair the neurodevelopment of full-term infants or those with birthweight within the normal range. Our sensitivity analysis excluding one study at a time showed that some exclusions caused the pooled estimates to be imprecise and some confidence intervals were no longer statistically significant. This might be due to the different set of confounders considered across studies, which could also lead to residual confounding.

The main strength of the present review was the inclusion of mostly cohort studies; only one was a case-control study that focused on delayed global neurodevelopment, thus, temporality between HDP and offspring’s neurodevelopment is not of concern. The review was conducted following the PRISMA guidelines, the quality of the selected studies was assessed with the NOS, and the certainty of the evidence was rated using the GRADE methodology. We were able to assess the effect of HDP on delayed global neurodevelopment as well as on separate domains of development including motor skills, cognitive, and language.

## Conclusion

Our results support the hypothesis that hypertensive disorders of pregnancy might increase the offspring’s risk of delayed neurodevelopment, specifically apparent in the motor and language domains. Such delay might be a consequence of being born preterm or with very low birthweight; thus, it is still unknown whether term infants with birthweight within the normal range are also affected in a similar fashion as preterm and low birthweight ones. Even though the evidence is moderate, prospective studies with longer follow-up times and careful control of confounders are needed to assess whether such neurodevelopmental alterations last at older ages, allowing decision makers to identify specific windows for interventions. HDP could be prevented to reduce the burden of its sequelae on the mother’s and offspring’s later health; therefore, a multidisciplinary approach from primary care providers and obstetricians is necessary; in addition, a close follow-up of the child’s neurodevelopment by pediatricians on regular healthcare visits could help identify any mild delay in a timely manner.

## Supplementary information

Supplementary material
