# Supplementary material for: Hypertensive disorders of pregnancy may increase the risk of neurodevelopmental delay in the offspring: a systematic review with meta-analysis
Source: Hypertens Res. 2026 Aug 3;49(10):2762–74. doi: 10.1038/s41440-026-02750-1 (PMC13634913; doi:10.1038/s41440-026-02750-1)
Supplement: Supplementary file 1 [file 41440_2026_2750_MOESM1_ESM.docx]

**Supplementary material**

**Hypertensive disorders of pregnancy may increase the risk of neurodevelopmental delay in the offspring: a systematic review with meta-analysis**

Leslie Marilu Zavala-Martínez,^a1^ Erika Marlen Hurtado-Salgado,^b,1^ Mary Carmen Baltazar-Reyes,^b^ Elizabeth Guzmán-Ortiz,^a^ Lea Aurora Cupul-Uicab^b^*

**Affiliations:**

^a^ Division of Health Sciences and Engineering, University of Guanajuato, Campus Celaya-Salvatierra. Av. Ing. Javier Barrios Sierra 201 Esq. Av. Baja California, Ejido de Santa María del Refugio, Celaya, Guanajuato, México. ZC 38140.

^b^ Center for Population Health Research, National Institute of Public Health. Avenida Universidad 655, Colonia Santa María Ahuacatitlan, Cuernavaca, Morelos, México. ZC 62100

^1^ These authors equally contributed.

***Corresponding author:**

Dr. Lea Aurora Cupul-Uicab

Population Health Research Center, National Institute of Public Health

Avenida Universidad 655 Sta. Ma. Ahuacatitlán

Cuernavaca, Morelos, México 62100

E-mail: lea.cupul@insp.mx; [acuicab@hotmail.com](mailto:acuicab@hotmail.com)

**Contents**

[**Supplementary Table 1.** Search algorithm used in the three databases.	4](#_Toc233038884)

[**Supplementary Table 2.** Eligibility criteria based on the PECOS statement.	5](#_Toc233038885)

[**Supplementary Table 3.** Quality assessment of the studies using the Newcastle-Ottawa Quality Scale for cohort and case-control designs.	6](#_Toc233038886)

[**Supplementary Table 4.** Subgroup meta-analysis according to age of the participants: hypertensive disorders of pregnancy and neurodevelopmental delay.	8](#_Toc233038887)

[**Supplementary Table 5.** Other sources of heterogeneity: meta-analysis of hypertensive disorders of pregnancy and neurodevelopmental delay.	9](#_Toc233038888)

[**Supplementary Table 6.** Sensitivity meta-analysis showing the effect on the pooled OR of excluding one study at a time: hypertensive disorders of pregnancy and global neurodevelopmental delay in the offspring.	10](#_Toc233038889)

[**Supplementary Table 7.** Sensitivity meta-analysis showing the effect on the pooled OR of excluding one study at a time: hypertensive disorders of pregnancy and motor neurodevelopmental delay in the offspring.	11](#_Toc233038890)

[**Supplementary Table 8.** Sensitivity meta-analysis showing the effect on the pooled OR of excluding one study at a time: hypertensive disorders of pregnancy and language development delay in the offspring.	12](#_Toc233038891)

[**Supplementary Table 9.** Sensitivity meta-analysis showing the effect on the pooled OR of excluding one study at a time: hypertensive disorders of pregnancy and cognitive developmental delay in the offspring.	13](#_Toc233038892)

[**Supplementary Table 10.** Meta-analysis of hypertensive disorders of pregnancy and global neurodevelopmental delay in the offspring: GRADE assessment.	14](#_Toc233038893)

[**Supplementary Table 11.** Meta-analysis of hypertensive disorders of pregnancy and motor neurodevelopmental delay in the offspring: GRADE assessment.	15](#_Toc233038894)

[**Supplementary Table 12.** Meta-analysis of hypertensive disorders of pregnancy and language developmental delay in the offspring: GRADE assessment.	16](#_Toc233038895)

[**Supplementary Table 13.** Meta-analysis of hypertensive disorders of pregnancy and cognitive neurodevelopmental delay in the offspring: GRADE assessment.	17](#_Toc233038896)

[**Supplementary Figure 1.** Meta-analysis of hypertensive disorders of pregnancy and gross motor skills developmental delay in the offspring.	18](#_Toc233038897)

[**Supplementary Figure 2.** Assessment of publication bias in meta-analyses of hypertensive disorders of pregnancy and neurodevelopmental delay in the offspring**.**	19](#_Toc233038898)

[**References**	23](#_Toc233038899)

# **Supplementary Table 1.** Search algorithm used in the three databases.

***PubMed, Web of Science, EBSCO:***

((“Neurodevelopment” OR “neurodevelopmental” OR "neurodevelopmental abnormalities" OR "neurodevelopmental assessment" OR "neurocognitive abnormalities" OR "neurocognitive assessment" OR “developmental assessment” OR "motor function" OR "motor alterations" OR "motor skills" OR "motor skills development" OR "motor skills assessment" OR "cognition development" OR "cognition assessment" OR "cognition alterations" OR "cognitive assessment" OR "cognitive alterations" OR "cognitive abnormalities" OR "language development" OR "language assessment" OR "communication skills assessment" OR "communication skills" OR "social communication skills" OR “social skills” OR “Ages and stages questionnaire” OR “Bayley scale”) AND (“pregnancy complications” OR “pregnancy high risk” OR “hypertension in pregnancy” OR "pregnancy hypertension" OR “pregnancy induced hypertension” OR “pregnancy related hypertension” OR “preeclampsia” OR “moderate preeclampsia” OR “severe preeclampsia” OR “proteinuria in pregnancy” OR “toxemia” OR “high risk pregnancy” OR “HELLP syndrome” OR “eclampsia”))

**Limits: from 2000 to 2024 (May 4^th^, 2024), including human studies published in English or Spanish.**

# **Supplementary Table 2.** Eligibility criteria based on the PECOS statement.

| **Population** | **Exposure** | **Comparators** | **Outcomes** | **Study design** |
| --- | --- | --- | --- | --- |
| Human population without restriction of sex, race, ethnicity, language, geography, or religion  Excluded those whose population included:  1. Products born from multiple pregnancies or those conceived through assisted reproduction.  2. Participants with chronic diseases.  3. Participants with a diagnosis of psychiatric disorders or intellectual disabilities. | Exposure to hypertensive disorders of pregnancy (HDP) with onset in the second half of pregnancy (>20 GA): including gestational hypertension, preeclampsia and eclampsia (with or without superimposed pre-pregnancy chronic hypertension).  HDP assessed as part of the study or through medical records (ICD), registries, or self-report.  Excluded studies that assess only pre-pregnancy chronic hypertension or HELLP syndrome. | Unexposed to hypertensive disorders of pregnancy, not exposed to chronic hypertension or HELLP syndrome. | Any problem, delay, or alteration in the offspring’s global neurologic development or in any domain: motor (gross or fine), cognitive, or language.  Outcomes ascertained through medical records, registries, assessment of neurologic development with screening tools, or guardian’s report.  Excluded studies that assessed intelligence quotient, academic performance, cerebral palsy, intellectual disability, and any psychiatric disorder as main outcomes. | Prospective or retrospective cohort studies, case-control studies nested within cohorts, and classic case-control studies.  Excludes cross-sectional studies, systematic reviews, meta-analyses, letters to the Editor and commentaries without original results, studies with sample size <10, case report, and publications with no peer review. |

Abbreviations: GA, Gestational age; HELLP, hemolysis, elevated liver enzymes, and low platelet count; ICD, international classification of diseases; PECOS, population, exposure, comparators, outcomes.

# **Supplementary Table 3.** Quality assessment of the studies using the Newcastle-Ottawa Quality Scale for cohort and case-control designs.

| Country (Author, year) | Selection  (4 stars) | Comparability  (2 stars) | Outcome or exposure  (3 stars) | Global score*  (9 stars) | Quality |
| --- | --- | --- | --- | --- | --- |
| Brazil (Silveira et al., 2007) | 4 | 1 | 3 | 8 | High |
| Italy (Spinillo et al., 2009) | 4 | 2 | 3 | 9 | High |
| Australia (Whitehouse et al., 2012) | 4 | 2 | 3 | 7 | High |
| Israel (Leitner et al., 2012) | 4 | 1 | 3 | 8 | High |
| Holland (Kerstjens et al., 2013) | 4 | 2 | 3 | 9 | High |
| Australia (Bolisetty et al., 2014) | 4 | 2 | 3 | 9 | High |
| Canada (Warshafsky et al., 2016) | 4 | 2 | 3 | 9 | High |
| India (Bharadwaj et al., 2018) | 4 | 1 | 3 | 8 | High |
| China (He et al., 2017) | 2 | 1 | 2 | 5 | Moderate |
| Finland (Girchenko et al., 2018) | 4 | 2 | 3 | 9 | High |
| Australia (Adane et al., 2018) | 3 | 2 | 3 | 8 | High |
| India (Bandiya et al., 2019) | 3 | 0 | 2 | 5 | Moderate |
| Japan (Noda et al., 2021) | 4 | 2 | 3 | 9 | High |
| Canada (Scime et al., 2021) | 4 | 2 | 3 | 9 | High |
| Israel (Manovitch et al., 2022) | 3 | 1 | 2 | 6 | Moderate |
| Argentina (Sala et al., 2022) | 4 | 2 | 3 | 9 | High |
| India (Koparkar et al., 2022) | 4 | 2 | 3 | 9 | High |
| USA (Palatnik et al., 2022) | 4 | 2 | 3 | 9 | High |
| South Korea (Kim et al., 2022) | 4 | 1 | 2 | 7 | High |
| Taiwan (Chang et al., 2023) | 4 | 2 | 3 | 9 | High |
| China (Huang et al., 2023) | 4 | 2 | 3 | 9 | High |

USA, United States of America

Quality of the studies according to the NOS global score: high, ≥7 stars; moderate, 4-6 stars; low, ≤3 stars.

# **Supplementary Table 4.** Subgroup meta-analysis according to age of the participants: hypertensive disorders of pregnancy and neurodevelopmental delay.

| **Subgroup defined by age at ND assessment for each outcome** | **Number of studies in each subgroup** | **Pooled OR**  **(95% CI)** | **Weight** | **I^2^ %** |
| --- | --- | --- | --- | --- |
| **Global neurodeveloment** |  |  |  |  |
| ≤24 months | 3/7 | 4.54 (0.31, 62.39) | 38.01 | 95.0% |
| >24 months | 4/7 | 1.48 (1.11, 1.97) | 61.99 | 24.4% |
| **Language development*** |  |  |  |  |
| ≤24 months | 4/6 | 2.22 (1.07, 4.61) | 61.35 | 63.9% |
| **Cognitive development*** |  |  |  |  |
| ≤24 months | 4/6 | 1.56 (0.79, 3.08) | 53.76 | 64.2% |

*There were not enough studies that assessed language and cognitive development at >24 months of age.

CI, confidence interval; ND, neurodevelopment; OR, odds ratio.

# **Supplementary Table 5.** Other sources of heterogeneity: meta-analysis of hypertensive disorders of pregnancy and neurodevelopmental delay.

| **Sources of heterogeneity for each outcome** | **Number of studies in each subgroup** | **Pooled OR**  **(95% CI)** | **Weight** | **I^2^ %** |
| --- | --- | --- | --- | --- |
| **Global neurodeveloment** |  |  |  |  |
| Definition of HDP excludes severe PE | 5/7 | 2.42 (1.02, 5.71) | 73.17 | 91.1% |
| ND assessed with ASQ | 4/7 | 1.48 (1.11, 1.97) | 61.99 | 24.4% |
| **Lenguaje development** |  |  |  |  |
| Definition of HDP excludes severe PE | 5/6 | 1.55 (0.90, 2.67) | 81.66 | 59.2% |
| ND assessed with Bayley-III | 4/6 | 2.23 (1.08, 4.62) | 61.35 | 63.9% |
| **Cognitive development** |  |  |  |  |
| Definition of HDP excludes severe PE | 5/6 | 1.14 (0.90, 1.45) | 91.22 | 0.0% |
| ND assessed with Bayley-III | 4/6 | 1.56 (0.79, 3.08) | 53.76 | 64.2% |

ASQ, ages and stages questionnaire; CI, confidence interval; ND, neurodevelopment; OR, odds ratio; PE, preeclampsia.

NOTE: There were not enough studies to conduct stratified meta-analysis according to the definition of HDP and according to the scale used to assess neurodevelopment, thus, we only presented the subgroups with at least three studies within strata to pool.

# **Supplementary Table 6.** Sensitivity meta-analysis showing the effect on the pooled OR of excluding one study at a time: hypertensive disorders of pregnancy and global neurodevelopmental delay in the offspring.

| **Excluded study** | **n** | **Country** | **Published**  **OR (95% CI)** | **Pooled OR**  **(95% CI)** | **I^2^ %** |
| --- | --- | --- | --- | --- | --- |
| ***No exclusions*** | **7214** |  |  | **2.07 (1.08, 3.95)** | ***86.9%*** |
| ***After the exclusions:*** | | | | | |
| Warshafsky et al., 2016 | 99 | Canada | 2.31 (0.63, 8.53) | 2.05 (1.02, 4.14) | 88.9% |
| Sala et al., 2022 | 132 | Argentina | 3.87 (1.26, 13.83) | 1.90 (0.95, 3.78) | 88.3% |
| **Spinillo et al., 2009** | **754** | **Italy** | **0.52 (0.32, 0.85)** | **2.68 (1.37, 5.26)** | **82.9%** |
| Kerstjens et al., 2013 | 834 | Holland | 1.23 (0.67, 2.28) | 2.37 (1.09, 5.17) | 89.1% |
| **He et al., 2017** | **1,337** | **China** | **54.40 (12.44, 237.88)** | **1.38 (0.85, 2.25)** | **76.3%** |
| Scime et al., 2021 | 1,554 | Canada | 1.29 (1.01, 1.65) | 2.53 (0.98, 6.56) | 89.1% |
| Girchenko et al., 2018 | 2,504 | Finland | 2.19 (1.30, 3.70) | 2.12 (0.97, 4.61) | 88.0% |

CI, confidence interval; OR, odds ratio.

# **Supplementary Table 7.** Sensitivity meta-analysis showing the effect on the pooled OR of excluding one study at a time: hypertensive disorders of pregnancy and motor neurodevelopmental delay in the offspring.

| **Excluded study** | **N** | **Country** | **Published**  **OR (95% CI)** | **Pooled OR**  **(95% CI)** | **I^2^ %** |
| --- | --- | --- | --- | --- | --- |
| ***No exclusions*** | **53138** |  |  | **1.25 (1.01, 1.55)** | **13%** |
| ***After the exclusions:*** | | | | | |
| **Silveira et al., 2007** | **86** | **Brazil** | **0.65 (0.25, 1.73)** | **1.30 (1.05, 1.60)** | **6.0%** |
| Sala et al., 2022 | 132 | Argentina | 3.01 (0.98, 10.64) | 1.23 (1.00, 1.51) | 4.7% |
| Chang et al., 2023 | 482 | Taiwan | 1.43 (0.71, 2.90) | 1.22 (0.95, 1.56) | 21.0% |
| Adane et al., 2018 | 771 | Australia | 0.89 (0.37, 2.13) | 1.27 (1.01, 1.60) | 17.0% |
| **Palatnik et al., 2022** | **1179** | **USA** | **0.70 (0.29, 1.68)** | **1.30 (1.05, 1.60)** | **6.6%** |
| Scime et al., 2021 | 1,554 | Canada | 1.29 (0.94, 1.76) | 1.21 (0.91, 1.61) | 21.7% |
| Girchenko et al., 2018 | 2,504 | Finland |  |  |  |
| ND motor grueso |  |  | 1.82 (0.91, 3.62) | 1.26 (1.02, 1.55) | 3.3% |
| ND motor fino |  |  | 0.51 (0.15, 1.69) |  |  |
| Huang et al., 2023 | 2,576 | China |  |  |  |
| ND motor grueso |  |  | 1.20 (0.67, 2.12) | 1.26 (0.96, 1.65) | 27.2% |
| ND motor fino |  |  | 0.86 (0.27, 2.77) |  |  |
| **Noda et al., 2021** | **43,854** | **Japan** | **1.69 (1.06, 2.70)** | **1.18 (0.94, 1.49)** | **8.1%** |

CI, confidence interval; OR, odds ratio.

# **Supplementary Table 8.** Sensitivity meta-analysis showing the effect on the pooled OR of excluding one study at a time: hypertensive disorders of pregnancy and language development delay in the offspring.

| **Excluded study** | **n** | **Country** | **Published**  **OR (95% CI)** | **Pooled OR**  **(95% CI)** | **I^2^ %** |
| --- | --- | --- | --- | --- | --- |
| ***No exclusions*** | **7581** |  |  | **1.87 (1.06, 3.28)** | **69%** |
| ***After the exclusions:*** | | | | | |
| Sala et al., 2022 | 132 | Argentina | 6.06 (1.15, 5.59) | 1.73 (0.97, 3.08) | 72.4% |
| **Chang et al., 2023** | **482** | **Taiwan** | **4.15 (1.99, 8.64)** | **1.54 (0.89, 2.66)** | **59.2%** |
| **Adane et al., 2018** | **708** | **Australia** | **0.92 (0.54, 1.57)** | **2.26 (1.31, 3.91)** | **52.3%** |
| Palatnik et al., 2022 | 1,179 | USA | 2.01 (1.24, 3.25) | 1.88 (0.87, 4.04) | 74.3% |
| Girchenko et al., 2018 | 2,504 | Finland | 2. 47 (1.10, 5.59) | 1.78 (0.91, 3.47) | 73.8% |
| Huang et al., 2023 | 2,576 | China | 0.76 (0.28, 2.10) | 2.17 (1.19, 3.94) | 69.8% |

CI, confidence interval; OR, odds ratio.

# **Supplementary Table 9.** Sensitivity meta-analysis showing the effect on the pooled OR of excluding one study at a time: hypertensive disorders of pregnancy and cognitive developmental delay in the offspring.

| **Excluded study** | **n** | **Country** | **Published**  **OR (95% CI)** | **Pooled OR**  **(95% CI)** | **I^2^ %** |
| --- | --- | --- | --- | --- | --- |
| ***No exclusions*** | **6,631** |  |  | **1.27 (0.88 1.82)** | ***48.5%*** |
| ***After the exclusions:*** | | | | | |
| **Adane et al., 2018** | **708** | **Australia** | **1.23 (0.85, 1.80)** | **1.40 (0.90, 2.16)** | **52.3%** |
| Scime et al., 2021 | 1,554 | Canada | 1.32 (0.88,1.99) | 1.30 (0.79, 2.14) | 57.9% |
| **Palatnik et al., 2022** | **1,179** | **USA** | **0.79 (0.44, 1.42)** | **1.40 (0.90, 2.16)** | **44.5%** |
| Sala et al., 2022 | 132 | Argentina | 2.82 (0.52, 23.76) | 1.23 (0.85, 1.80) | 55.4% |
| **Chang et al., 2023** | **482** | **Taiwan** | **4.38 (1.50, 12.71)** | **1.14 (0.89, 1.45)** | **0.0%** |
| Huang et al., 2023 | 2,576 | China | 1.34 (0.83, 2.16) | 1.29 (0.80, 2.08) | 58.0 % |

CI, confidence interval; OR, odds ratio.

# **Supplementary Table 10.** Meta-analysis of hypertensive disorders of pregnancy and global neurodevelopmental delay in the offspring: GRADE assessment.

| Number of studies | Quality assesment | | | | | | Summary of findings | |
| --- | --- | --- | --- | --- | --- | --- | --- | --- |
|  | **Study design** | **Risk of bias** | **Inconsistency** | **Indirectness** | **Imprecision** | **Publication bias** | **Relative effect** | **Certainty** |
| 7 | Prospective and retrospective^a^ | Not serious^b^ | Serious^c^ | Not serious^d^ | Not serious^e^ | No publication bias detected^f^ | 2.07  (1.08, 3.95) | (+++)  Moderate |

GRADE, Grading of Recommendations Assessment, Development, and Evaluation; HDP, hypertensive disease of pregnancy.

^a^ This meta-analysis is based on 7 studies, including six prospective cohorts and a case-control, which is retrospective by design.

^b^ All but one of the estimated ORs were adjusted for potential confounders. The scales to assess developmental delay are widely used and validated as screening tools, but non-differential misclassification cannot be disregarded. Given the design of the studies, the potential for selection bias is less likely.

^c^ Heterogeneity observed (I^2^=86.9%) in this meta-analysis suggests considerable inconsistency, which was partially explained by age of the participants. As shown in the stratified results, heterogeneity decreased (I^2^= 24.4%) across studies with participants older than 24 months by was greater when participants were 24 months of age or younger.

^d^ The definition of HDP included preeclampsia in all studies, but some also included eclampsia and gestational hypertension. And the screening scales used to define developmental delay varied across studies (*i.e.,* Baley II or III, ages, and stages questionnaire third edition, and Gesell’s developmental agenda).

^e^ The confidence interval of the pooled OR was somewhat wide, but the measure of effect suggests a strong association.

^f^ The Egger's test did not show statistically significant small-study effects (*p=0.22*), which was confirmed by the Begg’s test (*p=0.13*).

# **Supplementary Table 11.** Meta-analysis of hypertensive disorders of pregnancy and motor neurodevelopmental delay in the offspring: GRADE assessment.

| Number of studies | Quality assesment | | | | | | Summary of findings | |
| --- | --- | --- | --- | --- | --- | --- | --- | --- |
|  | **Study design** | **Risk of bias** | **Inconsistency** | **Indirectness** | **Imprecision** | **Publication bias** | **Relative effect** | **Certainty** |
| 9 | Prospective and retrospective^a^ | Not serious^b^ | Not serious^c^ | Serious^d^ | Not serious^e^ | No publication bias detected^f^ | 1.25  (1.01, 1.56) | (+++)  Moderate |

GRADE, Grading of Recommendations Assessment, Development, and Evaluation; HDP, hypertensive disease of pregnancy.

^a^ This meta-analysis was based on 9 cohort studies, 7 were prospective and 2 retrospective cohorts.

^b^ All but one of the estimated ORs were adjusted for potential confounders. The scales to assess developmental delay are widely used and validated as screening tools, but non-differential misclassification cannot be disregarded. Given the design of the studies, the potential for selection bias is less likely.

^c^ Heterogeneity observed (I^2^=13%) in this meta-analysis was not relevant, which does not support inconsistency across studies. No heterogeneity (I^2^= 0.0%) was observed when we conducted a meta-analysis of the three studies that assessed gross motor skills.

^d^ The definition of HDP included preeclampsia in all studies, but some also included eclampsia and gestational hypertension. The screening scales used to define delay in motor skills varied across studies and some did not differentiate between fine and gross motor skills.

^e^ The confidence interval of the pooled OR was somewhat wide, but the measure of effect suggests an association between HDP and offspring’s delay in motor skills.

^f^ The Egger's test did not show statistically significant small-study effects (*p=0.26*), which was confirmed by the Begg’s test (*p=0.12*).

# **Supplementary Table 12.** Meta-analysis of hypertensive disorders of pregnancy and language developmental delay in the offspring: GRADE assessment.

| Number of studies | Quality assesment | | | | | | Summary of findings | |
| --- | --- | --- | --- | --- | --- | --- | --- | --- |
|  | **Study design** | **Risk of bias** | **Inconsistency** | **Indirectness** | **Imprecision** | **Publication bias** | **Relative effect** | **Certainty** |
| 6 | Prospective and retrospective^a^ | Not serious^b^ | Serious^c^ | Not serious^d^ | Not serious^e^ | No publication bias detected^f^ | 1.87  (1.07, 3.29) | (+++)  Moderate |

GRADE, Grading of Recommendations Assessment, Development, and Evaluation; HDP, hypertensive disease of pregnancy.

^a^ This meta-analysis was based on 6 cohort studies, 5 were prospective and 1 retrospective cohorts.

^b^ All estimated ORs were adjusted for potential confounders. The scales to assess developmental delay are widely used and validated as screening tools, but non-differential misclassification cannot be disregarded. Given the design of the studies, the potential for selection bias is less likely.

^c^ Heterogeneity observed (I^2^=68.9%) in this meta-analysis suggests considerable inconsistency, which might be explained by age of the participants but was not properly assessed given the limited number of studies. Heterogeneity (I^2^=63.9%) decreased when the meta-analysis was limited to the studies with participants older than 24 months.

^d^ The definition of HDP included preeclampsia in all studies, but some also included eclampsia and gestational hypertension. And the screening scales used to define delay in language development varied across studies.

^e^ The confidence interval of the pooled OR was somewhat wide, but the measure of effect suggests a somewhat strong association between HDP and offspring’s delay in language development.

^f^ The Egger's test did not show statistically significant small-study effects (*p=0.59*), which was confirmed by the Begg’s test (*p=0.71*).

# **Supplementary Table 13.** Meta-analysis of hypertensive disorders of pregnancy and cognitive neurodevelopmental delay in the offspring: GRADE assessment.

| Number of studies | Quality assesment | | | | | | Summary of findings | |
| --- | --- | --- | --- | --- | --- | --- | --- | --- |
|  | **Study design** | **Risk of bias** | **Inconsistency** | **Indirectness** | **Imprecision** | **Publication bias** | **Relative effect** | **Certainty** |
| 6 | Prospective and retrospective^a^ | Not serious^b^ | Not serious^c^ | Not serious^d^ | Serious^e^ | No publication bias detected^f^ | 1.27  (0.88, 1.82) | (++)  Low |

GRADE, Grading of Recommendations Assessment, Development, and Evaluation; HDP, hypertensive disease of pregnancy.

^a^ This meta-analysis was based on 6 cohort studies, 5 were prospective and 1 retrospective cohorts.

^b^ All estimated OR were adjusted for potential confounders. The scales to assess cognitive delay are widely used and validated as screening tools, but non-differential misclassification cannot be disregarded. Given the design of the studies, the potential for selection bias is less likely.

^c^ Heterogeneity observed (I^2^=48.5%) in this meta-analysis suggests some inconsistency. Heterogeneity (I^2^= 64.2%) increased when the meta-analysis was limited to studies with participants older than 24 months.

^d^ The definition of HDP included preeclampsia in all studies, but some included eclampsia and gestational hypertension. And the screening scales used to define cognitive delay varied across studies.

^e^ The confidence interval of the pooled OR was wide and included the null value, even though the measure of effect suggests an association between HDP and offspring’s delay in cognitive development. These provided evidence of imprecision.

^f^ The Egger's test did not show statistically significant small-study effects (*p=0.30*), which was confirmed by the Begg’s test (*p=0.85*).

# **Supplementary Figure 1.** Meta-analysis of hypertensive disorders of pregnancy and gross motor skills developmental delay in the offspring.

CI, confidence interval; OR, odds ratio.

# **Supplementary Figure 2.** Assessment of publication bias in meta-analyses of hypertensive disorders of pregnancy and neurodevelopmental delay in the offspring**.**

A. Delayed global neurodevelopment.

B. Delayed motor development.

C. Delayed language development

D. Delayed cognitive development

# **References**

Adane, A. A., Mishra, G. D., & Tooth, L. R. (2018, Dec). Maternal preconception weight trajectories, pregnancy complications and offspring's childhood physical and cognitive development. *J Dev Orig Health Dis, 9*(6), 653-660. <https://doi.org/10.1017/S2040174418000570>

Bandiya, P., Datta, V., & Saili, A. (2019, Jun 15). Short Term Neurobehavioral Outcomes in Late Preterm Neonates Born to Pre-Eclamptic Mothers. *Indian Pediatr, 56*(6), 485-488. <https://www.ncbi.nlm.nih.gov/pubmed/31278229>

Bharadwaj, S. K., Vishnu Bhat, B., Vickneswaran, V., Adhisivam, B., Bobby, Z., & Habeebullah, S. (2018, May). Oxidative Stress, Antioxidant Status and Neurodevelopmental Outcome in Neonates Born to Pre-eclamptic Mothers. *Indian J Pediatr, 85*(5), 351-357. <https://doi.org/10.1007/s12098-017-2560-5>

Bolisetty, S., Dhawan, A., Abdel-Latif, M., Bajuk, B., Stack, J., Lui, K., New South, W., & Australian Capital Territory Neonatal Intensive Care Units' Data, C. (2014, Jan). Intraventricular hemorrhage and neurodevelopmental outcomes in extreme preterm infants. *Pediatrics, 133*(1), 55-62. <https://doi.org/10.1542/peds.2013-0372>

Chang, H. Y., Chen, C. P., Sun, F. J., & Chen, C. Y. (2023, Jun). Influence of pre-eclampsia on 2-year neurodevelopmental outcome of very-low-birth-weight infants. *Int J Gynaecol Obstet, 161*(3), 979-988. <https://doi.org/10.1002/ijgo.14614>

Girchenko, P., Tuovinen, S., Lahti-Pulkkinen, M., Lahti, J., Savolainen, K., Heinonen, K., Pyhala, R., Reynolds, R. M., Hamalainen, E., Villa, P. M., Kajantie, E., Pesonen, A. K., Laivuori, H., & Raikkonen, K. (2018, Jun). Maternal early pregnancy obesity and related pregnancy and pre-pregnancy disorders: associations with child developmental milestones in the prospective PREDO Study. *Int J Obes (Lond), 42*(5), 995-1007. <https://doi.org/10.1038/s41366-018-0061-x>

He, F., Li, Q.-P., Li, N.-P., Li, Y., Ma, X.-W., & Feng, Z.-C. (2017). Analysis of high-risk factors and effect of early intervention on preterm infant neurodevelopment. *Int J Clin Exp Med 10*(3), 9. <https://e-century.us/files/ijcem/10/3/ijcem0034549.pdf>

Huang, B., Wang, Y., Jiang, Y., Lv, H., Jiang, T., Qiu, Y., Lu, Q., Du, J., Lin, Y., & Ma, H. (2023, Sep 28). Association of maternal hypertensive disorders in pregnancy with infant neurodevelopment. *J Biomed Res, 37*(6), 479-491. <https://doi.org/10.7555/JBR.37.20230074>

Kerstjens, J. M., de Winter, A. F., Sollie, K. M., Bocca-Tjeertes, I. F., Potijk, M. R., Reijneveld, S. A., & Bos, A. F. (2013, Apr). Maternal and pregnancy-related factors associated with developmental delay in moderately preterm-born children. *Obstet Gynecol, 121*(4), 727-733. <https://doi.org/10.1097/AOG.0b013e3182860c52>

Kim, S. W., Youk, T., & Kim, J. (2022, Mar). Maternal and Neonatal Risk Factors Affecting the Occurrence of Neurodevelopmental Disorders: A Population-Based Nationwide Study. *Asia Pac J Public Health, 34*(2-3), 199-205. <https://doi.org/10.1177/10105395211066383>

Koparkar, S., Srivastava, L., Randhir, K., Dangat, K., Pisal, H., Kadam, V., Malshe, N., Wadhwani, N., Lalwani, S., Srinivasan, K., Kumaran, K., Fall, C., & Joshi, S. (2022, Apr). Cognitive function and behavioral problems in children born to mothers with preeclampsia: an Indian study. *Child Neuropsychol, 28*(3), 337-354. <https://doi.org/10.1080/09297049.2021.1978418>

Leitner, Y., Harel, S., Geva, R., Eshel, R., Yaffo, A., & Many, A. (2012, Nov). The neurocognitive outcome of IUGR children born to mothers with and without preeclampsia. *J Matern Fetal Neonatal Med, 25*(11), 2206-2208. <https://doi.org/10.3109/14767058.2012.684164>

Manovitch, Z., Morag, I., & Simchen, M. J. (2022, Mar). Neurodevelopmental outcomes of preterm infants born to preeclamptic mothers - A case-control study. *Eur J Obstet Gynecol Reprod Biol, 270*, 6-10. <https://doi.org/10.1016/j.ejogrb.2021.12.036>

Noda, M., Yoshida, S., Mishina, H., Matsubayashi, K., & Kawakami, K. (2021, Jun). Association between maternal hypertensive disorders of pregnancy and child neurodevelopment at 3 years of age: a retrospective cohort study. *J Dev Orig Health Dis, 12*(3), 428-435. <https://doi.org/10.1017/S2040174420000586>

Palatnik, A., Mele, L., Casey, B. M., Varner, M. W., Sorokin, Y., Reddy, U. M., Wapner, R. J., Thorp, J. M., Jr., Saade, G. R., Tita, A. T. N., Rouse, D. J., Sibai, B., Costantine, M. M., Mercer, B. M., Tolosa, J. E., Caritis, S. N., & Eunice Kennedy Shriver National Institute of Child Health Human Development Maternal-Fetal Medicine Units Network, B. M. D. U. S. A. (2022, Jul). Association between Hypertensive Disorders of Pregnancy and Long-Term Neurodevelopmental Outcomes in the Offspring. *Am J Perinatol, 39*(9), 921-929. <https://doi.org/10.1055/a-1692-0659>

Sala, M. E., Romero, M. F., Romero, A., Fasano, M. V., Varea, A. M., Carrera, P., Salazar, M. R., Espeche, W. G., & Gonzalez, H. F. (2022, Apr). Neurodevelopmental assessment of infants born to mothers with hypertensive disorder of pregnancy at six months of age. *J Dev Orig Health Dis, 13*(2), 197-203. <https://doi.org/10.1017/S204017442100026X>

Scime, N. V., Hetherington, E., Tomfohr-Madsen, L., Nettel-Aguirre, A., Chaput, K. H., & Tough, S. C. (2021). Hypertensive disorders in pregnancy and child development at 36 months in the All Our Families prospective cohort study. *PLoS One, 16*(12), e0260590. <https://doi.org/10.1371/journal.pone.0260590>

Silveira, R. C., Procianoy, R. S., Koch, M. S., Benjamin, A. C., & Schlindwein, C. F. (2007, Dec). Growth and neurodevelopment outcome of very low birth weight infants delivered by preeclamptic mothers. *Acta Paediatr, 96*(12), 1738-1742. <https://doi.org/10.1111/j.1651-2227.2007.00552.x>

Spinillo, A., Montanari, L., Gardella, B., Roccio, M., Stronati, M., & Fazzi, E. (2009, Jul). Infant sex, obstetric risk factors, and 2-year neurodevelopmental outcome among preterm infants. *Dev Med Child Neurol, 51*(7), 518-525. <https://doi.org/10.1111/j.1469-8749.2009.03273.x>

Warshafsky, C., Pudwell, J., Walker, M., Wen, S. W., Smith, G. N., & Preeclampsia New Emerging, T. (2016, Jul 7). Prospective assessment of neurodevelopment in children following a pregnancy complicated by severe pre-eclampsia. *BMJ Open, 6*(7), e010884. <https://doi.org/10.1136/bmjopen-2015-010884>

Whitehouse, A. J., Robinson, M., Newnham, J. P., & Pennell, C. E. (2012, Mar). Do hypertensive diseases of pregnancy disrupt neurocognitive development in offspring? *Paediatr Perinat Epidemiol, 26*(2), 101-108. <https://doi.org/10.1111/j.1365-3016.2011.01257.x>
